# Time for better time-restricted eating trials to lessen the burden of metabolic diseases

**DOI:** 10.1016/j.xcrm.2022.100665

**Published:** 2022-06-21

**Authors:** Pam R. Taub, Satchidananda Panda

**Affiliations:** 1 Division of Cardiovascular Medicine, Department of Medicine, University of California, San Diego, La Jolla, CA 92037, USA; 2 Regulatory Biology, the Salk Institute for Biological Studies, La Jolla, CA 92037, USA

## Abstract

Optimizing the quality, quantity, and timing of nutrition holds immense potential to improve health and prevent disease. The results of a recent randomized controlled trial^1^ have been widely misrepresented with the incorrect interpretation that optimizing the timing of food intake imparts no health benefits.

## Main text

Age-related cardiometabolic diseases constitute the major global burden of diseases.^2^ Therefore, scalable lifestyle interventions are necessary for their prevention. Calorie restriction (CR) by up to 25% to cause 5%–10% weight loss is the conventional approach to reduce obesity and its cardiometabolic complications. However, it is resource-intensive to implement and sustain long term.

Time-restricted eating (TRE) is a lowcost, sustainable lifestyle intervention approach to treat cardiometabolic diseases with or without weight loss. People in modern society eat over an extended period of ≥12 h.^3^ Such prolonged and erratic eating patterns in animal models disrupt circadian rhythms, which play an important role in metabolic homeostasis.^4^ TRE typically narrows the window of daily energy intake from ≥12 h to a consistent window of 8–10 h to improve circadian rhythms, usually without explicitly decreasing calories. Such an approach improves health in animal models and humans. In community-dwelling humans, TRE can inadvertently promote some CR,^3^ but cardiometabolic benefits are observed even among weight-stable individuals.^5^ As TRE is a new concept, most TRE studies are exploratory or smaller in size. Sufficiently powered randomized control trials are necessary to test TRE as a pragmatic lifestyle intervention; specifically, (1) the effect size of TRE on clinical outcomes with or without pharmacological or non-pharmacological interventions, (2) optimum strategy to facilitate the adoption of TRE, (3) long-term sustainability of TRE, and (4) return on investment in reducing cardiometabolic disease.

A recent report^1^ compared the effect of TRE + 25% CR versus 25% CR alone over 1 year in 139 obese participants. The primary outcome was a weight change. The CR + TRE group had −8 kg (95% CI; −9.6 to −6.4 kg) while the CR group had −6.3 kg (95% CI; −7.8 to −4.7 kg). There was no significant difference in weight loss between groups (−1.8 kg [95% CI; −4 to 0.4 kg, p = 0.11) or in body fat mass, visceral fat, systolic blood pressure, or diastolic blood pressure. This led the authors to conclude that “time-restricted eating was not more beneficial with regard to reduction in body weight, body fat, or metabolic risk factors than daily calorie restriction.”

Unfortunately, the study was not powered for the stated outcome: the difference in weight loss between TRE + CR and CR. The power calculation for the study was based on prior small studies that found weight loss between baseline and post-TRE in which participants reduced the eating window by ≥3 h and inadvertently reduced calories by 20%, and these studies lacked a CR-only group.^6^^,^^7^ Therefore, the study was not even powered to conclude that in healthy individuals with elevated BMI, with a ∼10-h eating window and no cardiometabolic abnormalities, there is no additional weight loss benefit in reducing the eating window by 2 h in comparison to 25% CR alone.

Moreover, the participants in this study were young, with a mean age of 32, and overall healthy, with the only abnormal metabolic parameter being an elevated BMI of 32. They had normal blood pressure, fasting glucose, HbA1c, and lipids. Their baseline eating window was already in the good range at ∼10 h, and for the study, they reduced their eating window to 8 h; such a modest reduction in eating window has rarely shown any health benefit.

The study was among the most rigorous CR study to date. They monitored daily food records from food pictures by two independent study staff; totaling >70,000 days of food records or a few hundreds of thousands of food pictures by manual inspection for energy estimation.^1^ The study also involved frequent interaction between the study team and the participants. The unprecedented resource-intensive nature of the study might have contributed to sustaining 25% CR at 12 months, but it also raises its utility as a scalable approach for CR. As the trial lacked a TRE-alone arm or a standard of care arm, it is hard to extrapolate these results to patients on a TRE-alone strategy.

These serious shortcomings of the study were lost, and the outcomes were generalized in the popular press with headlines stating, “Scientists Find no Benefit to Time Restricted Eating.” This has confused many patients with cardiometabolic disease who have been on TRE with great benefit. This is similar to when the United States Preventive Task Force released guidelines that aspirin was not recommended for primary prevention patients, and there were headlines such as “no benefit from aspirin.” This caused patients who were on aspirin for secondary prevention (history of myocardial infarction, coronary stents, stroke cardiac, bypass surgery) to stop their aspirin. Aspirin is indicated lifelong in these secondary prevention patients. The details of the study are important and get lost in the headlines.

TRE is a strategy that has a sound basis in animal and/or mechanistic studies, and it is starting to be translated into human studies. In animals on a healthy diet and TRE, cardiometabolic benefits are observed without significant weight loss.^8^ Similarly, in animal studies, CR + TRE animals live longer than their CR cohorts yet show no body weight difference.^9^ In our pilot study of patients with metabolic syndrome, where the average eating window reduced from 15.13 ± 1.13 h to 10.78 ± 1.18 h, we found significant improvement in cardiometabolic parameters such as atherogenic lipids, HbA1C, and blood pressure that were disproportionately larger than weight loss.^10^ Altogether, studies that objectively monitor adherence to TRE have found cardiometabolic benefits among at-risk individuals, and the benefits can be independent of weight loss.

We have ongoing randomized control studies (NCT NCT04057339 and NCT05365529) to assess the impact of TRE on patients with metabolic syndrome and type 2 diabetes. Such studies on populations with cardiometabolic disease are needed to determine the clinical benefit of TRE. As with any pharmacologic or device therapy, there is a “sweet spot” of return on investment where patients get the most benefit. It is likely that TRE is most beneficial in populations where there are underlying cardiometabolic derangements.

## References

[1] Liu D., Huang Y., Huang C., Yang S., Wei X., Zhang P., Guo D., Lin J., Xu B., Li C. (2022). Calorie restriction with or without time-restricted eating in weight loss. N. Engl. J. Med..

[2] Murray C.J.L., Aravkin A.Y., Zheng P., Abbafati C., Abbas K.M., Abbasi-Kangevari M., Abd-Allah F., Abdelalim A., Abdollahi M., Abdollahpour I. (2020). Global burden of 87 risk factors in 204 countries and territories, 1990-2019: a systematic analysis for the Global Burden of Disease Study 2019. Lancet.

[3] Manoogian E.N., Chow L.S., Taub P.R., Laferrere B., Panda S. (2022). Time-restricted eating for the prevention and management of metabolic diseases. Endocr. Rev..

[4] Panda S. (2016). Circadian physiology of metabolism. Science.

[5] Sutton E.F., Beyl R., Early K.S., Cefalu W.T., Ravussin E., Peterson C.M. (2018). Early time-restricted feeding improves Insulin Sensitivity, blood pressure, and Oxidative stress even without weight loss in men with Prediabetes. Cell Metabol..

[6] Gill S., Panda S. (2015). A Smartphone App Reveals erratic Diurnal eating patterns in humans that can Be Modulated for health benefits. Cell Metabol..

[7] Gabel K., Hoddy K.K., Haggerty N., Song J., Kroeger C.M., Trepanowski J.F., Panda S., Varady K.A. (2018). Effects of 8-hour time restricted feeding on body weight and metabolic disease risk factors in obese adults: a pilot study. Nutr. Healthy Aging.

[8] Hatori M., Vollmers C., Zarrinpar A., DiTacchio L., Bushong E.A., Gill S., Leblanc M., Chaix A., Joens M., Fitzpatrick J.A. (2012). Time-restricted feeding without reducing caloric intake prevents metabolic diseases in mice fed a high-fat diet. Cell Metabol..

[9] Acosta-Rodriguez V., Rijo-Ferreira F., Izumo M., Xu P., Wight-Carter M., Green C.B., Takahashi J.S. (2022). Circadian alignment of early onset caloric restriction promotes longevity in male C57BL/6J mice. Science.

[10] Wilkinson M.J., Manoogian E.N.C., Zadourian A., Lo H., Fakhouri S., Shoghi A., Wang X., Fleischer J.G., Navlakha S., Panda S., Taub P.R. (2020). Ten-hour time-restricted eating reduces weight, blood pressure, and atherogenic lipids in patients with metabolic syndrome. Cell Metabol..

